# Supplementary material for: Artemisinin Attenuated Hydrogen Peroxide (H2O2)-Induced Oxidative Injury in SH-SY5Y and Hippocampal Neurons via the Activation of AMPK Pathway
Source: Int J Mol Sci. 2019 May 31;20(11):2680. doi: 10.3390/ijms20112680 (PMC6600327; doi:10.3390/ijms20112680)
Supplement: Supplementary file 1 [file ijms-20-02680-s001.pdf]

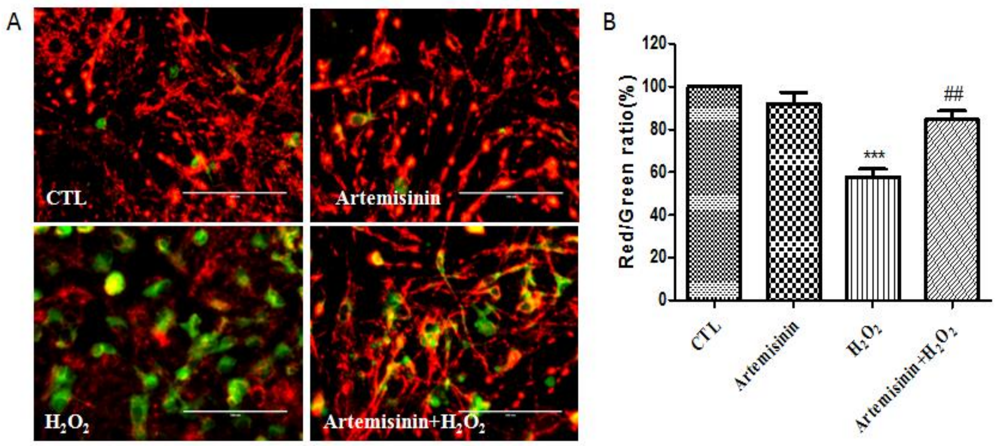

Figure S1. Artemisinin attenuated H_2O_2 -induced mitochondrial membrane potential (△ψm) loss in the hippocampal neurons. (A) After pre-treatment with 25 μM Artemisinin for 2 h, cells were incubated with or without 100 μM H_2O_2 for another 24 h, △ψm was determined by the JC-1 assay. (B) Quantification of (A). The data was represented as the mean ± SD. ***P < 0.001 versus control group; ##P < 0.01 versus H_2O_2 -treated group were considered significantly different.

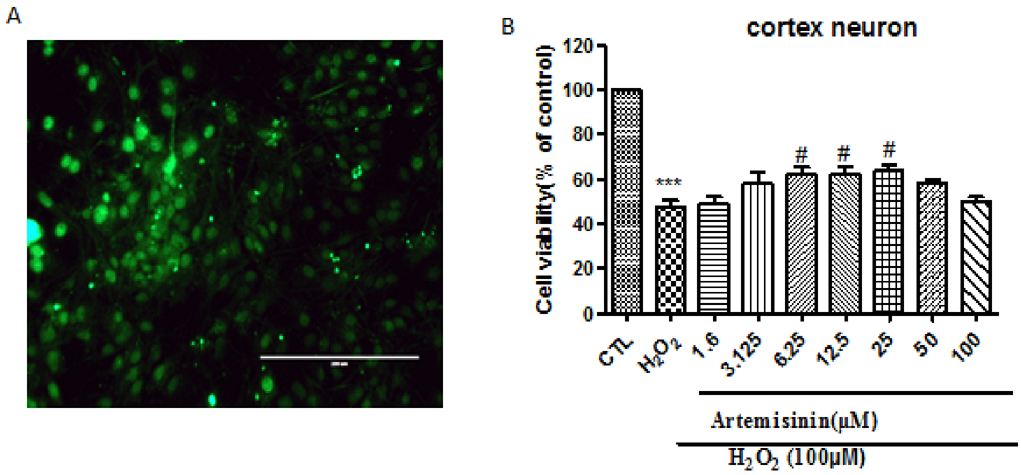

Figure S2. Artemisinin attenuated the decrease in cell viability caused by H_2O_2 in cortex neurons. (A) Immunocytochemistry of NeuN. (B).Cells were pretreated with artemisinin at indicated concentrations and then induced with or without 100μM H_2O_2 for a further 24h and cell viability was measured using the MTT assay. ***P < 0.001 versus control group; #P < 0.05versus H_2O_2 -treated group were considered significantly different.

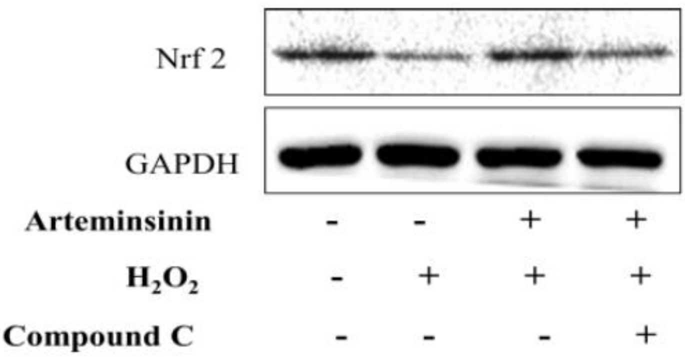

Figure S3 Artemisinin s increased expression levels of Nrf2 in the SH-SY5Y cells. Cells were pre-treated

with 5 μM Compound C (AMPK inhibitor) for 30 min, and treated with 12.5 μM artemisinin for 2 hours, then incubated with or without 600 μM H2O2 for another 24 h, The expression of Nrf2 was measured by western blot.

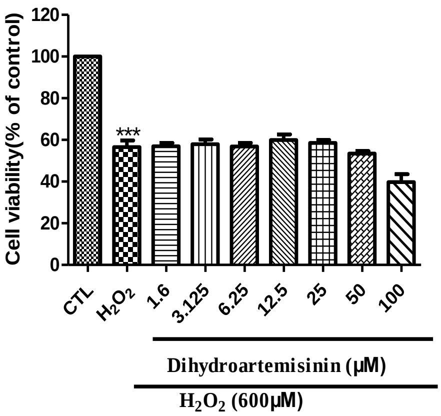

Figure S4.Dihydroartemisinin (DHA) had no significant protective effects under the same conditions as artemisinin. Cells were pretreated with artemisinin at indicated concentrations and then induced with or without 100μM H_2O_2 for a further 24h and cell viability was measured using the MTT assay. ***$P < 0.001$ versus control group.
